# Severe pantothenic acid deficiency induces alterations in the intestinal mucosal proteome of starter Pekin ducks

**DOI:** 10.1186/s12864-021-07820-x

**Published:** 2021-06-30

**Authors:** Jing Tang, Yulong Feng, Bo Zhang, Yongbao Wu, Zhanbao Guo, Suyun Liang, Zhengkui Zhou, Ming Xie, Shuisheng Hou

**Affiliations:** 1 State Key Laboratory of Animal Nutrition, Key Laboratory of Animal (Poultry) Genetics Breeding and Reproduction, Ministry of Agriculture and Rural Affairs, Institute of Animal Sciences, Chinese Academy of Agricultural Sciences, Beijing, 100193 China; 2 Guizhou Animal Husbandry and Veterinary Research Institute, Guizhou Academy of Agricultural Sciences, Guiyang, 550000 Guizhou China

**Keywords:** Pantothenic acid deficiency, Intestinal hypofunction, Mucosal proteomics, Hypoglycemia, Actin cytoskeleton

## Abstract

**Background:**

Pantothenic acid deficiency (PAD) results in growth depression and intestinal hypofunction of animals. However, the underlying molecular mechanisms remain to be elucidated. Mucosal proteome might reflect dietary influences on physiological processes.

**Results:**

A total of 128 white Pekin ducks of one-day-old were randomly assigned to two groups, fed either a PAD or a pantothenic acid adequate (control, CON) diet. After a 16-day feeding period, two ducks from each replicate were sampled to measure plasma parameters, intestinal morphology, and mucosal proteome. Compared to the CON group, high mortality, growth retardation, fasting hypoglycemia, reduced plasma insulin, and oxidative stress were observed in the PAD group. Furthermore, PAD induced morphological alterations of the small intestine indicated by reduced villus height and villus surface area of duodenum, jejunum, and ileum. The duodenum mucosal proteome of ducks showed that 198 proteins were up-regulated and 223 proteins were down-regulated (> 1.5-fold change) in the PAD group compared to those in the CON group. Selected proteins were confirmed by Western blotting. Pathway analysis of these proteins exhibited the suppression of glycolysis and gluconeogenesis, fatty acid beta oxidation, tricarboxylic acid cycle, oxidative phosphorylation, oxidative stress, and intestinal absorption in the PAD group, indicating impaired energy generation and abnormal intestinal absorption. We also show that nine out of eleven proteins involved in regulation of actin cytoskeleton were up-regulated by PAD, probably indicates reduced intestinal integrity.

**Conclusion:**

PAD leads to growth depression and intestinal hypofunction of ducks, which are associated with impaired energy generation, abnormal intestinal absorption, and regulation of actin cytoskeleton processes. These findings provide insights into the mechanisms of intestinal hypofunction induced by PAD.

**Supplementary Information:**

The online version contains supplementary material available at 10.1186/s12864-021-07820-x.

## Background

Pantothenic acid is a precursor of two coenzymes, coenzyme A (CoA) and acyl-carrier-protein. The coenzymes of this vitamin participate in various metabolic reactions, such as glucose, fatty acids and amino acids entering into energy-yielding tricarboxylic acid (TCA) cycle, fatty acid oxidation and synthesis, cholesterol synthesis, acetylcholine synthesis, and heme synthesis etc. [1, 2]. Its importance is highlighted by the adverse effects of pantothenic acid deficiency (PAD) in mammals such as rats, cats, and pigs, including growth depression, skin lesions, diarrhea, loss of hair [2–6]. Also in poultry studies, PAD results in growth retardation, poor feathering, dermatosis, and high mortality in chicks, turkeys, geese, and ducks [7–13]. It has been demonstrated extensively that pantothenic acid can keep the structure of intestine integrity and maintain the intestinal function of animals [3, 14–16]. Intestinal hypofunction was shown to be a major consequence of PAD in rats, dogs, cats, chicks, and fish, such as intestinal ulceration, diarrhea, and colitis [4, 5, 17–21]. Rats deficient in pantothenic acid exhibit duodenitis and duodenal ulcers [18], as well as duodenal changes including eventual atrophy of crypts, diminution in size of villi [16]. In addition, previous studies in fish showed that PAD decreased intestinal digestive and absorptive capacities by reducing the activities of both intestinal brush border enzymes and digestive enzymes [14, 21].

In addition, pantothenic acid can protect cell membrane against peroxidative damage by increasing glutathione content [22–24]. Previous studies have shown that dietary PAD could lead to oxidative stress in geese [9] and fish [21]. It is proposed that oxidative stress induced by PAD leads to intestinal injury and hypofunction. So far, the detailed mechanisms of growth depression and intestinal hypofunction due to PAD are still unclear. In order to understand the underlying mechanisms, we established a PAD duck model, identified an overview of underlying processes and the extend of alterations of intestinal mucosa using a proteomic approach.

## Results

### Mortality and growth performance

The mortality of PAD ducks was greater than the control (CON) birds (*P* < 0.001; Table 1). In comparison with the CON group, average daily weight gain (ADG) and average daily feed intake (ADFI) were declined in the PAD group, while feed conversion ratio (FCR) was increased (*P* < 0.001; Table 1).

**Table 1:** Growth performance on day 16 of ducks in the pantothenic acid-deficient (PAD) and control (CON) group

Variable	PAD	CON	SEM	*P*-value
Mortality (%)	68.7^a^	0^b^	3.16	< 0.001
ADG (g/d)	10.6^b^	31.3^a^	1.13	< 0.001
ADFI (g/d)	16.8^b^	40.1^b^	1.30	< 0.001
FCR (g:g)	1.66^a^	1.28^b^	0.03	< 0.001

ADG, average daily weight gain; ADFI, average daily feed intake; FCR, feed conversion ratio; and SEM, standard error of the mean

^a, b^ Mean values with unlike superscript letters were significantly different (*P* < 0.05). Data were analyzed by the Student’s *t* test. Data are expressed as mean and SEM (*n* = 8)

### Plasma parameters

Plasma pantothenic acid concentration decreased by 85%, while plasma alkaline phosphatase (ALP) activity decreased by approximately 70% as a result of PAD when compared to the CON group (*P* < 0.001; Table 2). Compared to the CON group, plasma glucose and insulin contents were declined in the PAD group (*P* < 0.01; Table 2). Plasma glucagon did not differ between the PAD and CON group (*P* > 0.05; Table 2). Compared to the CON group, plasma malondialdehyde (MDA) content was increased, while plasma total superoxide dismutase (T-SOD) activity was decreased in the PAD group (*P* < 0.001; Table 2).

**Table 2:** Plasma parameters of 16-day-old ducks in the pantothenic acid-deficient (PAD) and control (CON) group

Variable	PAD	CON	SEM	*P*-value
Pantothenic acid (nmol/L)	121^b^	800^a^	54.5	< 0.001
Glucose (mmol/L)	7.33^b^	9.91^a^	0.14	< 0.001
ALP (U/L)	236^b^	740^a^	27.7	< 0.001
Insulin (μIU/mL)	6.11^b^	8.84^a^	0.38	0.008
Glucagon (pg/mL)	147	147	5.72	0.971
MDA	6.47^a^	4.18^b^	0.21	< 0.001
T-SOD	80.2^b^	103^a^	2.24	0.020

ALP, alkaline phosphatase; MDA, malondialdehyde; T-SOD, total superoxide dismutase; SEM, standard error of the mean

^a, b^ Mean values with unlike superscript letters were significantly different (*P* < 0.05). Data were analyzed by the Student’s *t* test. Data are expressed as mean and SEM (*n* = 8)

### Intestinal morphology analyses

Dietary PAD resulted in morphological alterations of the small intestine of Pekin ducks. Compared to the CON group, PAD reduced villus height and villus surface area of duodenum, jejunum, and ileum (*P* < 0.001; Table 3). Villus width and crypt depth of both duodenum and jejunum were not affected by PAD in ducks (*P* > 0.05; Table 3). Compared to the CON group, PAD decreased crypt depth of ileum in ducks (*P* < 0.001; Table 3) but not villus width of ileum (*P* > 0.05; Table 3).

**Table 3:** Intestinal mucosal histomorphology of 16-day-old ducks in the pantothenic acid-deficient (PAD) and control (CON) group

Items	PAD	CON	SEM	*P*-value
Duodenum				
Villus height (μm)	666^b^	893^a^	27.7	< 0.001
Villus width (μm)	108	130	5.60	0.093
Crypt depth (μm)	175	214	9.90	0.153
Villus surface area (mm^2^)	0.23^b^	0.36^a^	0.018	0.001
Jejunum				
Villus height (μm)	467^b^	678^a^	42.4	0.033
Villus width (μm)	121	134	7.20	0.249
Crypt depth (μm)	123	158	8.30	0.076
Villus surface area (mm^2^)	0.18^b^	0.28^a^	0.019	0.010
Ileum				
Villus height (μm)	305^b^	534^a^	32.4	< 0.001
Villus width (μm)	118	122	3.40	0.618
Crypt depth (μm)	126^b^	194^a^	10.5	0.002
Villus surface area (mm^2^)	0.11^b^	0.20^a^	0.014	< 0.001

SEM, standard error of the mean

^a, b^ Mean values with unlike superscript letters were significantly different (*P* < 0.05). Data were analyzed by the Student’s *t* test. Data are expressed as mean and SEM (*n* = 8)

### Changes in the intestinal mucosal proteomics of duck in response to PAD

A total of 22,973 peptide belonging to 3345 proteins were identified in the duodenum mucosa of two groups. Comparisons of the relative abundance of proteins from mucosa of PAD ducks with those of CON ducks showed that a total of 421 proteins showed a fold change (FC) > 1.5, of which 198 proteins were up-regulated and 223 proteins were down-regulated. The complete list of proteins altered by PAD is presented in Additional file 1.

**Fig. 1:**
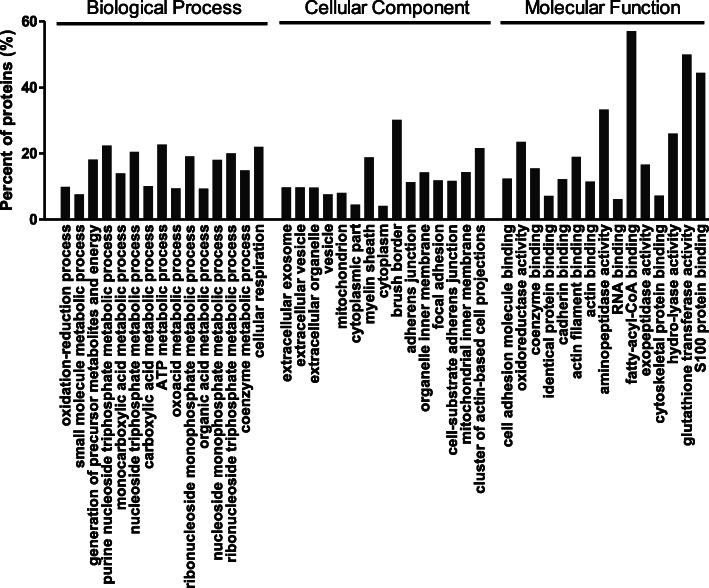
Top 15 significantly terms enriched biological processes, cellular components, and molecular functions

All the differentially expressed proteins in mucosa in response to PAD were used to conduct GO categories of biological process, cellular component, and molecular function, and pathway analysis. As shown in Fig. 1, the top 15 enriched terms under biological process included oxidation-reduction process, small molecule metabolic process, generation of precursor metabolites and energy, purine nucleoside triphosphate metabolic process, monocarboxylic acid metabolic process, nucleoside triphosphate metabolic process, carboxylic acid metabolic process, ATP metabolic process, oxoacid metabolic process, ribonucleoside monophosphate metabolic process, organic acid metabolic process, nucleoside monophosphate metabolic process, ribonucleoside triphosphate metabolic process, coenzyme metabolic process, and cellular respiration. The top 15 enriched terms under cellular component included extracellular exosome, extracellular vesicle, extracellular organelle, vesicle, mitochondrion, cytoplasmic part, myelin sheath, cytoplasm, brush border, adherens junction, organelle inner membrane, focal adhesion, cell-substrate adherens junction, mitochondrial inner membrane, and cluster of actin-based cell projections. The top 15 enriched terms under molecular function included cell adhesion molecule binding, oxidoreductase activity, coenzyme binding, identical protein binding, cadherin binding, actin filament binding, actin binding, aminopeptidase activity, RNA binding, fatty-acyl-CoA binding, exopeptidase activity, cytoskeletal protein binding, hydro-lyase activity, glutathione transferase activity, and S100 protein binding.

**Fig. 2:**
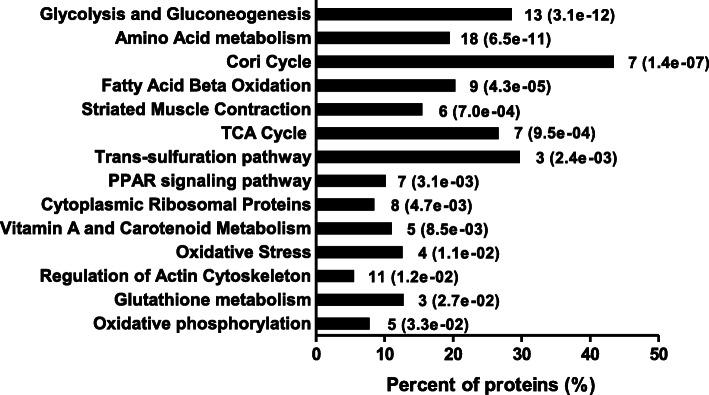
The pathway analysis by KEGG on differentially expressed proteins

Based on the pathway analysis by Kyoto Encyclopedia of Genes and Genomes (KEGG), the significantly affected pathways were glycolysis and gluconeogenesis, amino acid metabolism, cori cycle, fatty acid beta oxidation, striated muscle contraction, TCA cycle, trans-sulfuration pathway, PPAR signaling pathway, cytoplasmic ribosomal proteins, vitamin A and carotenoid metabolism, oxidative stress, regulation of actin cytoskeleton, glutathione metabolism, and oxidative phosphorylation (Fig. 2). Among them, energy metabolism (glycolysis and gluconeogenesis, fatty acid beta oxidation, TCA cycle, and oxidative phosphorylation), regulation of actin cytoskeleton, and oxidative stress were selected in the present study and the proteins involved in these processes are listed in Table 4.

**Table 4:** Selected differentially expressed proteins in duodenum mucosa caused by pantothenic acid deficiency

UniProtKB ID	Protein name	Short name	Fold change^*^	*P*-Value
**Glycolysis and gluconeogenesis**				
U3IHG8	Fructose-bisphosphate aldolase	ALDOB	−5.47	5.90E-09
U3IVG9	Hexokinase domain containing 1	HKDC1	−4.34	1.13E-13
U3IR52	Alpha-enolase	ENO1	−3.75	6.19E-08
U3J2H8	Fructose-bisphosphatase 1	FBP1	−2.42	1.35E-10
U3J1L1	Glyceraldehyde-3-phosphate dehydrogenase	GAPDH	−3.02	9.49E-06
U3I0F9	Pyruvate kinase	PKM	−3.28	1.94E-07
U3IZA5	ATP-dependent 6-phosphofructokinase	PFKP	−2.33	3.93E-05
U3I8D8	Triosephosphate isomerase	TPI1	−2.93	9.45E-05
U3ILF5	Phosphoglycerate kinase	PGK1	−2.01	1.71E-06
U3IR48	Dihydrolipoyl dehydrogenase	DLD	−1.66	2.09E-02
U3IEW2	Pyruvate dehydrogenase E1 beta subunit	PDHB	− 1.58	1.79E-02
U3I939	Fructose-bisphosphate aldolase	ALDOA	1.89	1.34E-03
U3IR68	Hexokinase 1	HK1	3.31	1.12E-02
**Fatty acid beta oxidation**				
U3J4Z9	Acyl-CoA synthetase long chain family member 5	ACSL5	−3.06	6.74E-09
U3I9A1	Acyl-CoA dehydrogenase family member 11	ACAD11	−1.96	1.34E-02
U3ITA9	Medium-chain specific acyl-CoA dehydrogenase	ACADM	−1.85	1.79E-05
U3IHS8	Carnitine O-acetyltransferase	CRAT	−1.78	6.54E-03
U3IR48	Dihydrolipoyl dehydrogenase	DLD	−1.66	2.09E-02
U3I6S1	Hydroxyacyl-CoA dehydrogenase trifunctional multienzyme complex subunit beta	HADHB	−1.65	1.60E-03
U3IEF4	Enoyl-CoA delta isomerase 2	ECI2	−1.65	4.61E-02
U3J928	Acyl-coenzyme A oxidase	ACOX1	−1.51	1.68E-05
U3IDQ1	Acyl-coenzyme A oxidase	ACOX2	−1.74	3.50E-05
**TCA cycle**				
U3IR48	Dihydrolipoyl dehydrogenase	DLD	−1.66	2.09E-02
R0J775	Aconitase 1 (Fragment)	ACO1	−1.64	2.75E-10
U3IC15	Aconitate hydratase, mitochondrial	ACO2	−2.13	9.22E-11
R0JXM5	Malate dehydrogenase (Fragment)	MDH1	−1.78	2.25E-05
U3IA60	Malate dehydrogenase	MDH2	−2.89	4.45E-08
U3IEW2	Pyruvate dehydrogenase E1 beta subunit	PDHB	−1.58	1.79E-02
U3J597	Isocitrate dehydrogenase [NADP]	IDH1	1.58	5.05E-06
**Oxidative phosphorylation**				
U3J532	NADH:ubiquinone oxidoreductase subunit A5	NDUFA5	−1.58	1.94E-02
R0LLX6	NADH dehydrogenase [ubiquinone] 1 alpha subcomplex subunit 6 (Fragment)	NDUFA6	−1.56	4.97E-02
U3J175	ATP synthase, H+ transporting, mitochondrial Fo complex subunit B1	ATP5F1	−1.80	2.12E-03
R0LYJ7	ATP synthase subunit d, mitochondrial (Fragment)	ATP5H	−1.79	2.69E-04
R0LIL9	ATP synthase subunit O, mitochondrial (Fragment)	ATP5O	−1.60	2.16E-03
**Regulation of actin cytoskeleton**				
U3IFN5	Villin 1	VIL1	−3.12	1.13E-08
U3IY96	Ezrin	EZR	−2.00	1.61E-05
U3IRY0	Vimentin	VIM	2.66	5.54E-04
U3J6G2	Tropomyosin 3	TPM3	4.51	1.36E-02
U3I7J8	Tropomyosin alpha-1 chain	TPM1	9.58	6.76E-04
U3IA79	Myosin light chain 1	MYL1	11.47	2.50E-06
R0LM85	Myosin light chain kinase (Fragment)	MYLK	2.88	1.83E-02
U3I4I9	Actinin alpha 1	ACTN1	2.13	5.89E-07
U3IZ83	Fibronectin 1	FN1	2.28	4.31E-02
U3IR26	Vinculin	VCL	1.87	4.54E-11
U3I935	Moesin	MSN	2.07	5.48E-03
**Oxidative stress**				
U3J0T0	Amine oxidase	MAOA	−2.56	5.04E-08
A0A172QNN4	Catalase	CAT	−1.84	1.49E-05
R0JZP2	Microsomal glutathione S-transferase 1 (Fragment)	MGST1	−1.57	2.66E-02
U3I5T1	Glutamate-cysteine ligase catalytic subunit	GCLC	1.57	9.62E-03
**Intestinal absorption**				
U3IRP7	Solute carrier family 2 member 2	SLC2A2	−5.11	3.17E-02
U3IFN5	Villin 1	VIL1	−3.12	1.13E-08
U3IY96	Ezrin	EZR	−2.00	1.61E-05
U3IUS6	Monoacylglycerol O-acyltransferase 2	MOGAT2	−2.28	5.35E-03

*TCA,* tricarboxylic acid

* Fold change is expressed as the ratio of the pantothenic acid-deficient to the control group. For the down-regulated proteins, the fold change was transformed to the corresponding negative value

Of the proteins enriched in glycolysis and gluconeogenesis, eleven proteins were down-regulated (fructose-bisphosphate aldolase (ALDOB), hexokinase domain containing 1 (HKDC1), alpha-enolase (ENO1), fructose-bisphosphatase 1 (FBP1), glyceraldehyde-3-phosphate dehydrogenase (GAPDH), pyruvate kinase (PKM), ATP-dependent 6-phosphofructokinase (PFKP), triosephosphate isomerase (TPI1), phosphoglycerate kinase (PGK1), dihydrolipoyl dehydrogenase (DLD), and pyruvate dehydrogenase E1 beta subunit (PDHB)), while two proteins were up-regulated (fructose-bisphosphate aldolase (ALDOA) and hexokinase 1 (HK1)).

Nine proteins involved in fatty acid beta oxidation were all down-regulated in the PAD group, including acyl-CoA synthetase long chain family member 5 (ACSL5), acyl-CoA dehydrogenase family member 11 (ACAD11), medium-chain specific acyl-CoA dehydrogenase (ACADM), carnitine O-acetyltransferase (CRAT), DLD, hydroxyacyl-CoA dehydrogenase trifunctional multienzyme complex subunit beta (HADHB), enoyl-CoA delta isomerase 2 (ECI2), acyl-coenzyme A oxidase (ACOX1), and acyl-coenzyme A oxidase (ACOX2).

Of the proteins enriched in TCA cycle, six proteins were down-regulated (DLD, aconitase 1 (Fragment) (ACO1), aconitate hydratase, mitochondrial (ACO2), malate dehydrogenase (fragment) (MDH1), malate dehydrogenase (MDH2), and pyruvate dehydrogenase E1 beta subunit (PDHB)), while one protein was up-regulated (isocitrate dehydrogenase [NADP] (IDH1)).

Five proteins were involved in the oxidative phosphorylation, which were all down-regulated in the PAD group (NADH:ubiquinone oxidoreductase subunit A5 (NDUFA5), NADH dehydrogenase [ubiquinone] 1 alpha subcomplex subunit 6 (Fragment) (NDUFA6), ATP synthase, H+ transporting, mitochondrial Fo complex subunit B1 (ATP5F1), ATP synthase subunit d, mitochondrial (Fragment) (ATP5H), and ATP synthase subunit O, mitochondrial (Fragment) (ATP5O)).

Of the proteins involved in regulation of actin cytoskeleton, two proteins were down-regulated (Villin 1 (VIL1) and Ezrin (EZR)), while nine proteins were up-regulated (vimentin (VIM), tropomyosin 3 (TPM3), tropomyosin alpha-1 chain (TPM1), myosin light chain 1 (MYL1), myosin light chain kinase (Fragment) (MYLK), actinin alpha 1 (ACTN1), fibronectin 1 (FN1), vinculin (VCL), and moesin (MSN)).

Of the proteins involved in oxidative stress, three proteins were all down-regulated (amine oxidase (MAOA), catalase (CAT), and microsomal glutathione S-transferase 1 (Fragment) (MGST1)), while one protein was up-regulated (glutamate-cysteine ligase catalytic subunit (GCLC)).

Besides, we found that four proteins were involved in intestinal absorption, which were all down-regulated by PAD, including solute carrier family 2 member 2 (SLC2A2), VIL1, EZR, and monoacylglycerol O-acyltransferase 2 (MOGAT2).

### Western blot analyses

**Fig. 3:**
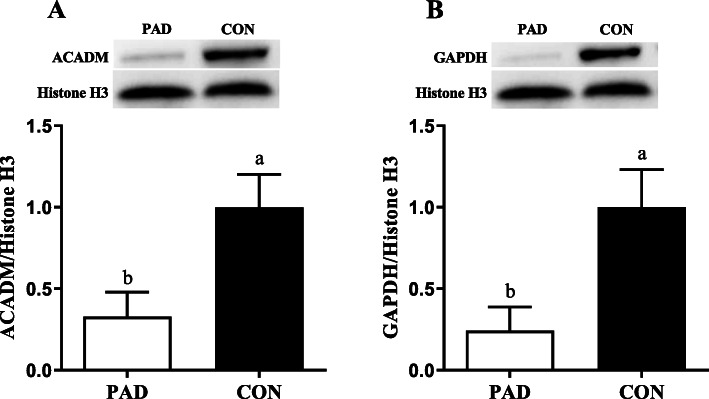
Western blot analysis of medium-chain-specific acyl-CoA dehydrogenase (ACADM; A) and glyceraldehyde-3-phosphate dehydrogenase (GAPDH; B) protein abundance of mucosal tissue of ducks in the pantothenic acid deficient (PAD) and Control (CON) groups. Loading control, histone H3, was used to normalize the levels of ACADM and GAPDH. Representative Western blots are shown. The images of complete Western blots are shown in Additional file 2. Values are means with their standard errors. ^a, b^ Mean values with unlike letters were significantly different (*P* < 0.05). Data were analyzed by the Student’s *t* test (*n* = 4)

To validate the isobaric tags for relative and absolute quantification (iTRAQ) results, the abundance of ACADM and GAPDH were analyzed by Western blot. Compared to the CON group, the protein expressions of ACADM and GAPDH were decreased in the PAD group (Fig. 3), which were consistent with the iTRAQ results.

## Discussion

Previous studies reported that dietary PAD causes deficiency symptoms such as growth retardation, dermatosis, diarrhea, and even death in both mammals [2–6] and poultry [7–13, 19, 25]. In ducks, growth retardation and excessive exudate from the eyes were exhibited in response to PAD [13]. Consistent with previous studies, the PAD ducks showed growth depression, exudate on eyelids, poor feathering, and a high mortality rate in the present study. Furthermore, poor pantothenic acid status was observed in PAD ducks indicated by reduced plasma pantothenic acid concentration, which is a sensitive biomarker for pantothenic acid status [21, 26, 27]. These results indicate that a severe PAD animal model was successfully established.

It has been shown that pantothenic acid can maintain intestinal function by keeping the structure of intestine integrity of animals [3, 14–16]. Insufficient of pantothenic acid would cause intestinal hypofunction in rats, dogs, cats, chicks and fish [4, 5, 17–21]. And a feature common to experimental PAD in different animals is intestinal ulceration [4, 5, 17–20]. Duodenitis and duodenal ulcers [18], as well as duodenal changes including eventual atrophy of crypts, diminution in size of villi, were observed in PAD rats [16]. In agreement with the previous studies, we observed small intestine morphological changes indicated by the reduced villus height and villus surface area in PAD ducks in the present study, indicating damage to the intestinal epithelium.

Furthermore, PAD leads to abnormalities in carbohydrate metabolism. Low fasting blood glucose levels and increased sensitivity to insulin were found in PAD rats and dogs [28–32]. In agreement with previous studies, PAD caused fasting hypoglycemia and decreased plasma insulin level in ducks in the present study, indicating abnormal glucose metabolism.

Together, dietary PAD of ducks resulted in growth retardation, alterations of intestinal morphology and function, and abnormal glucose metabolism. However, limited data are available currently concerning the molecular mechanisms behind. Therefore, we used a proteomic approach, for the first time, to investigate the metabolic disorder of small intestine induced by PAD to explain intestinal hypofunction and growth depression. Proteomic analysis revealed 421 differentially expressed proteins in the mucosa of PAD ducks compared to CON birds, indicating an important impact of pantothenic acid on intestinal function. The differentially expressed proteins are mainly enriched in glycolysis and gluconeogenesis, fatty acid beta oxidation, oxidative phosphorylation, TCA cycle, intestinal absorption, regulation of actin cytoskeleton, and oxidative stress. These processes probably underlie the intestinal hypofunction and growth depression induced by PAD. Notably, the CoA-binding proteins were reduced in the intestinal mucosa of PAD ducks, such as ACSL5, ACAD11, ACADM, HADHB, ECI2, ACOX1, and ACOX2. This finding supports the hypothesis that CoA-binding proteins may be depressed as a result of dietary PAD.

### Glycolysis and gluconeogenesis

A total of thirteen proteins participating in the glycolysis and gluconeogenesis pathway were altered by PAD. Of these, twelve proteins were involved in glycolysis, two enhanced (ALDOA and HK1) and ten diminished (ALDOB, HKDC1, ENO1, GAPDH, PKM, PFKP, TPI1, PGK1, DLD, and PDHB). HKDC1 and HK1 are two isozymes of hexokinases, which mediate the initial step of glycolysis by catalyzing phosphorylation of D-glucose to D-glucose 6-phosphate [33]. PFKP catalyzes the phosphorylation of fructose 6-phosphate to fructose 1,6-bisphosphate by ATP [34]. ALDOA and ALDOB are two isoforms of aldolase family which are located in skeletal muscle and liver tissue respectively, cleaves fructose-1,6-bisphosphate to triose phosphates [35]. TPI1 catalyzes the interconversion between dihydroxyacetone phosphate and D-glyceraldehyde-3-phosphate [36]. GAPDH catalyzes the conversion of glyceraldehyde 3-phosphate into 1,3-diphosphoglycerate [37]. PGK1 catalyzes one of the two ATP producing reactions in the glycolytic pathway via the reversible conversion of 1,3-diphosphoglycerate to 3-phosphoglycerate [38]. ENO1 catalyzes the formation of phosphoenolpyruvate from 2-phosphoglycerate [39]. PKM catalyzes the second ATP generation reaction in the glycolytic pathway via conversion of phosphoenolpyruvate to pyruvate [40]. PDHB is a subunit of pyruvate dehydrogenase (E1). As the E3 component of pyruvate dehydrogenase complex, DLD oxidizes dihydrolipoic acid to lipoic acid [41]. Pyruvate dehydrogenase complex converts pyruvate to acetyl-CoA [42, 43]. Therefore, ten out of twelve proteins (ALDOB, HKDC1, ENO1, GAPDH, PKM, PFKP, TPI1, PGK1, DLD, and PDHB) were down-regulated in the PAD group suggests that the rate of glycolysis in the intestinal mucosa may be impaired. Besides, one protein (FBP1) involved in gluconeogenesis process was down-regulated by PAD, indicating a depressed gluconeogenesis. FBP1, a rate-limiting enzyme in gluconeogenesis, catalyzes the hydrolysis of fructose 1,6-bisphosphate to fructose 6-phosphate.

### Fatty acid beta oxidation

In the present study, PAD down-regulated 9 proteins involved in fatty acid beta oxidation, including ACSL5, ACAD11, ACADM, CRAT, DLD, HADHB, ECI2, ACOX1, and ACOX2. Notably, among these, ACSL5, ACAD11, ACADM, HADHB, ECI2, ACOX1, and ACOX2 are belong to CoA-binding proteins, which were all reduced due to PAD. ACSL5 belongs to the acyl-CoA synthetase family, catalyzing free fatty acids into fatty acyl-CoA esters, which play key roles in lipid biosynthesis and fatty acid degradation [44]. ACAD11 and ACADM are two members of fatty acyl-CoA dehydrogenases that catalyze the first step in each cycle of fatty acid beta oxidation [45]. CRAT catalyzes the reversible transfer of an acetyl group from acyl-CoA to carnitine [46]. ECI2 is involved in the beta oxidation of unsaturated fatty acids, catalyzing the formation of 2-trans-enoyl-CoA from 3-cis or trans-enoyl-CoA [47]. HADHB catalyzes the final step of beta-oxidation, in which 3-ketoacyl CoA is cleaved by the thiol group of another molecule of Coenzyme A [48]. ACOX1 and ACOX2 catalyze the first and rate-limiting step of peroxisomal fatty acid beta oxidation, the desaturation of acyl-CoAs to 2-trans-enoyl-CoAs. ACOX1 catalyzes medium to very long straight-chain fatty acids [49], while ACOX2 catalyze the CoA-esters of very long-chain fatty acids, branched-chain fatty acids and the C27-bile acid intermediates [50]. The down-regulation of all these proteins involved in the fatty acid beta oxidation process in PAD ducks may indicate that this process is impaired. This implication is supported by previous finding in rats that PAD reduced CoA and short-chain acyl-CoA contents [51], as well as hepatic peroxisomal fatty acid beta oxidation [6].

### TCA cycle

PAD down-regulated six proteins involved in the TCA cycle, including DLD, ACO1, ACO2, MDH1, MDH2, and PDHB, and up-regulated one protein, IDH1. PDHB is a subunit of pyruvate dehydrogenase (E1). As the E3 component of pyruvate dehydrogenase complex and α-ketoglutarate dehydrogenase complex, DLD oxidizes dihydrolipoic acid to lipoic acid [41]. Pyruvate dehydrogenase complex converts pyruvate to acetyl-CoA [42, 43]. α-ketoglutarate dehydrogenase catalyzes the oxidation of α-ketoglutarate to form succinyl-CoA, which is a rate-limiting enzyme of the TCA cycle [52, 53]. ACO1 and ACO2 are two isozymes of aconitate hydratase located in cytoplasmic and mitochondrial respectively, which catalyzes the isomerization of citrate to isocitrate via cis-aconitate. MDH1 and MDH2 are two isozymes of malate dehydrogenase located in cytoplasmic and mitochondrial respectively, which catalyzes the reversible interconversion of malate and oxaloacetate [54]. IDH1 is a subunit of isocitrate dehydrogenase, catalyzing the oxidative decarboxylation of isocitrate into α-ketoglutarate [55]. Six out of seven proteins (DLD, ACO1, ACO2, MDH1, MDH2, and PDHB) were down-regulated due to PAD, indicating a decreased TCA cycle.

### Oxidative phosphorylation

PAD down-regulated 5 proteins involved in oxidative phosphorylation, including NDUFA5, NDUFA6, ATP5F1, ATP5H, and ATP5PO. NDUFA5 and NDUFA6, the subunits of complex I, are involved in complex I assembly [56, 57]. ATP5F1, ATP5H, and ATP5PO, the subunits of complex V, are involved in complex V assembly [58–60]. The down-regulated expression of proteins involved in oxidative phosphorylation, probably indicates that this process is impaired by PAD.

Together, our duodenum mucosal proteomic analysis revealed glycolysis and gluconeogenesis, fatty acid beta oxidation, TCA cycle, and oxidative phosphorylation are probably impaired in response to PAD, consequently resulting in insufficient energy generation in the small intestine and subsequent growth retardation.

### Regulation of actin cytoskeleton

Eleven proteins altered by PAD have actin-binding domains and play direct roles in the organization of structure of the cytoskeleton, including two down-regulated proteins (VIL1 and EZR) and nine up-regulated proteins (ACTN1, FN1, MSN, MYL1, MYLK, VCL, TPM3, TPM1, and VIM). VIL1 and EZR (also known as villin 2; VIL2) are microvillar proteins in intestinal epithelial cells [61]. VIL1 is an epithelial cell-specific actin-binding protein that regulates cell migration, cell death, cell morphology, and epithelial-to-mesenchy-mal transition [62, 63]. EZR is critical for the de novo lumen formation and expansion during villus morphogenesis, and EZR absence resulted in abnormal villus morphogenesis [64, 65].

Actinin is a component of stress fibers and links the cytoskeleton to adherens-type junctions. As one isoform of actinin, ACTN1 plays a major role in cell migration and adhesion [66]. Besides, ACTN1 can directly binds to VCL, and the two proteins cooperate to organize the cytoskeleton at adhesion junctions [67]. FN1, a glycoprotein component of the extracellular matrix, has a role in cell adhesion and migration [68]. VIM induces changes in cell shape, motility, and adhesion during the epithelial to mesenchymal transition [69]. MYLK induces contraction of the perijunctional actomyosin ring through myosin II regulatory light chain phosphorylation, and thereby increases intestinal epithelial permeability when activated [70]. TPM3 and TPM1 are two isoforms of tropomyosins which play important roles in the regulation of assembly, stability, and motility of the intestinal epithelial cells [71]. Collectively, the over expression of nine proteins (ACTN1, FN1, MSN, MYL1, MYLK, VCL, TPM3, TPM1, and VIM) and down-regulation of two proteins (VIL1 and EZR) in response to PAD probably indicates orchestrated regulation of actin cytoskeleton dynamics and the negative impact on intestinal integrity. Remodeling of the cytoskeleton is fundamental in proliferation, apoptosis, cell invasion and metastasis [72]. Therefore, these altered proteins involved in regulation of actin cytoskeleton in response to PAD probably resulted in morphological changes of small intestine, such as atrophy of their intestinal villus. Furthermore, it is reported that ATP depletion uncouples the gate and fence functions of the tight junction and induces actin network dissolution of epithelial cells [73]. The alterations of intestinal morphology and actin cytoskeleton due to PAD in the present study may be attributed to ATP depletion indicated by impaired glycolysis, fatty acid beta oxidation, TCA cycle, and oxidative phosphorylation processes.

### Oxidative stress

PAD down-regulated three proteins involved in the oxidative stress, including MAOA, CAT, and MGST1, and up-regulated one protein, GCLC. MAOA metabolizes dopamine to dihydroxyphenylacetic acid and H_2_O_2,_ a potential source of reactive oxygen species [74]. Both CAT and MGST1 take part in the oxidative stress defense as its scavenging of H_2_O_2_ [75, 76]. GCLC is a subunit of glutamate cysteine ligase which catalyzes the rate-limiting step in reduced glutathione (GSH) synthesis. And glutamate cysteine ligase is often activated to increase cellular GSH content in response to oxidative stress [77]. Collectively, the reduction of MAOA, CAT, and MGST1 in PAD ducks, as well as the enhanced GCLC, indicates small intestinal oxidative stress was induced. This is supported by the results of increased plasma MDA content and decreased T-SOD activity in the present study and is consistent with previous studies in geese [9] and fish [21]. It has been shown that pantothenic acid protects cells against oxidative stress by promoting glutathione levels and cellular repair mechanisms [22–24]. Furthermore, PAD induced oxidative stress in ducks, which may be associated with intestinal injury and morphological alterations.

### Intestinal absorption

A novel and important finding of this study is that four proteins (SLC2A2, VIL1, EZR, and MOGAT2) involved in intestinal absorption were down-regulated in duodenum mucosa as a result of PAD. Intestinal SLC2A2 (GLUT2) is known as a means to transfer glucose and fructose from the lumen to the bloodstream and, thereby, to provide sugar to tissue. And intestinal SLC2A2 deletion in mice induced glucose malabsorption visualized by the delay in the distribution of oral sugar in tissues, as well as decreased microvillus length and body weight gain [78]. It is reported pantothenic acid appears to be part of a glucose carrier system [79], therefore, PAD may direct reduce SLC2A2 and resulted in abnormal glucose absorption and hypoglycemia in ducks. Villin (VIL1 and EZR) is also involved in the absorptive and secretory function of epithelial cells by modulating F-actin polymerization/depolymerization. Specifically, villin-depleted mice showed a reduction in intestinal glucose absorption [80]. Decreased protein expression of SLC2A2, VIL1, and EZR due to PAD probably impair glucose absorption system, which may provide a possible explanation for fasting hypoglycemia that is seen. MOGAT2 plays a central role in absorption of dietary fat in the small intestine by catalyzing the resynthesis of triacylglycerol in enterocytes [81]. MGAT2 deficient specifically in the small intestine showed a delay in fat absorption in mice [82]. Therefore, a reduction of SLC2A2, VIL1, EZR, and MOGAT2 suggests that glucose and fat malabsorption in the small intestine may be induced by PAD, which probably leads to growth depression observed. This is in line with the previous findings in fish that PAD decreased the digestive and absorptive capacities indicated by the reduced the activities of both intestinal brush border enzymes and digestive enzymes [14, 21].

## Conclusion

PAD caused growth retardation, fasting hypoglycemia, morphological alterations of the small intestine, and oxidative stress in ducks. We have performed a proteomic profiling further to investigate the effects of PAD on duodenum mucosal proteome of ducks. The results indicated that PAD may suppress energy generation processes such as glycolysis and gluconeogenesis, fatty acid beta oxidation, TCA cycle, and oxidative phosphorylation, leading to impaired ATP generation. Furthermore, PAD may induce glucose and fat malabsorption in the small intestine indicated by four diminished proteins involved. Besides, PAD probably leads to negative impacts on intestinal integrity and morphology because most of proteins involved in regulation of actin cytoskeleton were up-regulated. To be sum, PAD causes intestinal hypofunction and growth depression probably by impairing glycolysis and gluconeogenesis, fatty acid beta oxidation, TCA cycle, oxidative phosphorylation, actin cytoskeleton, and intestinal absorption processes. These findings add to our understanding of the mechanisms of intestinal hypofunction due to PAD.

## Methods

### Animals ethics statement

All experimental procedures with ducks were performed according to the Guidelines for Experimental Animals established by the Ministry of Science and Technology (Beijing, China). Ethical approval on animal survival was given by the animal ethics committee of the Institute of Animal Sciences (IAS), Chinese Academy of Agricultural Sciences (CAAS, Beijing, China) with the following reference number: IASCAAS-2019–19.

### Animals and housing

A total of 128 one-day-old male white Pekin ducks (*Anas platyrhynchos*) were assigned randomly to one of two dietary treatments of 8 replicate pens with 8 birds per pen. The ducks were either fed with a PAD diet or a pantothenic acid adequate (control, CON) diet. From hatch to 16 days of age, all ducks were reared in pens in a temperature-controlled room with feed and water ad libitum. And the light was continuously on.

### Diet

The pantothenic acid-deficient basal diet was formulated in line with NRC (1994) [83], containing 4.65 mg pantothenic acid /kg of diet (Table 5). To produce the PAD diet and control diet, the basal diets were supplemented with 0 and 8 mg/kg of crystalline calcium pantothenate respectively (purity, 99%; Xinfu Technology Co. Ltd., Hangzhou, China). The pantothenic acid concentration for the control diet (12.65 mg/kg) met the recommendation provided by NRC (1994) [83].

**Table 5:** Composition of pantothenic acid-deficient basal diet from hatch to 16 days of age (% as-fed)

Item	Value
Ingredient, %	
Corn	79.7
Soy isolate protein	16.0
Limestone	1.0
Dicalcium phosphate	1.6
Vitamin and trace mineral premix^a^	1.0
Sodium chloride	0.3
DL-Methionine	0.3
L-Lysine·HCl	0.1
Total	100.0
Calculated composition	
Metabolizable energy^b^, MJ/kg	13.35
Crude protein	20.39
Calcium	0.93
Nonphytate phosphorus	0.43
Lysine	1.17
Methionine	0.57
Methionine + cysteine	0.80
Threonine	0.77
Tryptophan	0.19
Arginine	1.38
Pantothenic acid^c^, mg/kg	4.65

^a^ Supplied per kilogram of total diet: Cu (CuSO_4_•5H_2_O), 10 mg; Fe (FeSO_4_•7H_2_O), 60 mg; Zn (ZnO), 60 mg; Mn (MnSO_4_•H_2_O), 80 mg; Se (NaSeO_3_), 0.3 mg; I (KI), 0.2 mg; choline chloride, 1000 mg; vitamin A (retinyl acetate), 10,000 IU; vitamin D_3_ (Cholcalciferol), 3000 IU; vitamin E (DL-α-tocopheryl acetate), 20 IU; vitamin K_3_ (menadione sodium bisulfate), 2 mg; thiamin (thiamin mononitrate), 2 mg; riboflavin, 10 mg; pyridoxine hydrochloride, 4 mg; cobalamin, 0.02 mg; nicotinic acid, 50 mg; folic acid, 1 mg; biotin, 0.2 mg

^b^ The value is calculated according to the AME of ducks (Ministry of Agriculture of China, 2012)

^c^ The value was based on high performance liquid chromatography coupled with triple quadrupole mass spectrometry

### Sampling

At 16 days of age, after overnight fasting, duck weight and feed intake from each pen were recorded to calculate ADG, ADFI, and FCR. ADFI and FCR were all corrected for mortality. Two ducks from each pen were randomly selected for sampling as follows. Blood was taken via jugular vein to harvest plasma, stored at − 20 °C until further analysis. Thereafter, these selected ducks were sacrificed by CO_2_ inhalation and intestinal tissues were obtained immediately. The intestinal sections were divided into the duodenum, jejunum, and ileum. And they were rinsed with physiological saline and then cut into 1 cm length segments, fixed in 10% neutral formalin used for histological analysis. Duodenum mucosa from the remaining segment was obtained as described previously [84], snap frozen in liquid nitrogen, and stored at − 80 °C until further analysis.

### Pantothenic acid content

Feed and plasma pantothenic acid concentrations were measured by HPLC (Agilent 1290) coupled with triple quadrupole mass spectrometry (Agilent 6470) according to the methods described previously [85]. Before LC/MS analysis, feed and plasma samples were pretreated according to the methods described previously [86, 87]. The peak was identified by the pure authentic standards (Sigma-Aldrich, St. Louis, MO, USA).

### Plasma parameters

Plasma glucose and ALP were determined by commercial kits following the manufacturer’s instructions (BioSino Bio-technology and Science Inc., Beijing, China). Plasma MDA, T-SOD, insulin, and glucagon were measured by commercial kits following the manufacturer’s protocols (Nanjing Jiancheng Institute of Bioengineering, Nanjing, Jiangsu, China).

### Intestinal morphology assessment

The duodenum, jejunum, and ileum sections were embedded in paraffin and transversely sectioned in (4 μm thick) and stained with hematoxylin and eosin following deparaffinization and dehydration. Intestinal tissues and structures were observed using a BH2 Olympus microscope (Olympus, Tokyo, Japan) and analyzed using an image analysis system (Olympus 6.0). Villus height, villus width, crypt depth, and villus surface area were assessed following the method as described previously [84].

### Duodenum mucosal proteomics

Three individual duodenum mucosa samples were randomly chosen from each group to conduct the iTRAQ assays. Proteins were extracted and digested as described previously [88]. The digested samples were labelled following the manufacturer’s protocols with an 8-plex iTRAQ kit (AB SCIEX, Foster City, USA). The PAD samples were labelled with iTRAQ tags 113, 114, and 115, while the CON samples were labelled with tags 116, 117, and 118. The labelled samples were combined and fractionated into 20 fractions by HPLC (DINOEX Ultimate 3000 BioRS, Thermo Fisher, Waltham, MA, USA). We performed LC-electrospray ionization-MS/MS analysis on a Triple TOF 5600 plus system (AB SCIEX, Framingham, USA). The collected raw MS/MS data were searched according to the Uniprot-Swissport *Anas platyrhynchos* Database *UniProt_Mallard_8839* using ProteinPilot Software (version 4.5, AB SCIEX). To minimize false-positive results, we counted only peptides at the 95% CI and a false discovery rate < 0.01, containing at least one unique peptide. For protein quantitation, a differentially expressed protein containing at least two unique spectra was used with a FC value > 1.5 or < − 1.5 with *P* < 0.05 between the PAD and CON groups. For functional annotation, the differentially expressed proteins induced by PAD were performed KEGG pathway and Gene Ontology (GO) enrichment analysis using ClueGo software as described previously [88].

### Western blot analyses

Western blot analysis of two proteins, ACADM and GAPDH, were performed following the method as described [89]. Primary antibodies (1 μg/ml) against ACADM (ab92461; Abcam) and GAPDH (HX1828; Huaxingbio) were used. Histone H3 (A2348; ABclonal) served as a loading control.

### Statistical analyses

For results of growth performance, plasma parameters, and intestinal mucosal histomorphology, data were analyzed using the Student’s *t* test procedures of SAS software (SAS Institute Inc., 2011). The replicate pen of 8 ducks for growth performance or two ducks for other indices served as the experimental unit. The variability in the data was expressed as the standard error of the means (SEM). Differences between means were considered statistically significant at *P* < 0.05.

## Supplementary Information

**Additional file 1.** List of differentially expressed proteins in duodenum mucosa caused by pantothenic acid deficiency.

**Additional file 2 Fig. S1.** Complete images of Western blots shown in Fig. 3. Western blot analysis of medium-chain-specific acyl-CoA dehydrogenase (ACADM) and glyceraldehyde-3-phosphate dehydrogenase (GAPDH) protein abundance of mucosal tissue of ducks in the pantothenic acid deficient (PAD) and Control (CON) groups. Histone H3 served as a loading control.

## Data Availability

The data sets supporting the conclusions of this article are included within the article and its additional file. The mass spectrometry proteomics data have been deposited to the ProteomeXchange Consortium via the PRIDE (https://www.ebi.ac.uk/pride/archive/) partner repository with the data set identifier PXD026710.

## Abbreviations

ADG: average daily weight gain
ADFI: average daily feed intake
ALP: alkaline phosphatase
CoA: coenzyme A
CON: control
FC: fold change
FCR: feed conversion ratio
GO: Gene Ontology
HPLC: high performance liquid chromatography
iTRAQ: isobaric tags for relative and absolute quantification
KEGG: Kyoto Encyclopedia of Genes and Genomes
MDA: malondialdehyde
PAD: pantothenic acid deficiency
SEM: standard error of the mean
TCA: tricarboxylic acid
T-SOD: total superoxide dismutase
