# Supplementary material for: Severe pantothenic acid deficiency induces alterations in the intestinal mucosal proteome of starter Pekin ducks
Source: BMC Genomics. 2021 Jun 30;22:491. doi: 10.1186/s12864-021-07820-x (PMC8246668; doi:10.1186/s12864-021-07820-x)
Supplement: Supplementary file 1 — Additional file 1. List of differentially expressed proteins in duodenum mucosa caused by pantothenic acid deficiency. [file 12864_2021_7820_MOESM1_ESM.docx]

Additional file 1**.** List of differentially expressed proteins in mucosa caused by pantothenic acid deficiency*.*

| UniProtKB ID | Protein name | Short name | Fold change^*^ | *P*-Value |
| --- | --- | --- | --- | --- |
| U3J0V1 | Uncharacterized protein | N/A | -6.37 | 1.25E-07 |
| U3IHG8 | Fructose-bisphosphate aldolase | ALDOB | -5.47 | 5.90E-09 |
| R0KYP7 | Calbindin (Fragment) | Anapl_14984 | -5.23 | 4.86E-11 |
| U3IRP7 | Solute carrier family 2 member 2 | SLC2A2 | -5.11 | 3.17E-02 |
| U3IRA6 | Beta-carotene oxygenase 1 | BCO1 | -5.01 | 6.63E-08 |
| U3IX73 | N-acyl phosphatidylethanolamine phospholipase D | NAPEPLD | -5.00 | 3.41E-03 |
| U3II97 | Solute carrier family 11 member 2 | SLC11A2 | -4.90 | 2.85E-02 |
| U3IPB4 | Uncharacterized protein | N/A | -4.88 | 0.00E+00 |
| U3IEW1 | Galectin | LGALS3 | -4.69 | 3.03E-05 |
| R0K265 | Epoxide hydrolase 1 (Fragment) | Anapl_05710 | -4.69 | 2.30E-06 |
| U3IVG9 | Hexokinase domain containing 1 | HKDC1 | -4.34 | 1.13E-13 |
| R0L0P0 | Creatine kinase B (Fragment) | CKB | -4.14 | 4.51E-06 |
| U3J8L3 | Solute carrier family 25 member 13 | SLC25A13 | -4.06 | 5.50E-10 |
| U3IR52 | Alpha-enolase | ENO1 | -3.75 | 6.19E-08 |
| R0LPY1 | Protocadherin-24 (Fragment) | Anapl_14302 | -3.46 | 7.99E-03 |
| U3IK39 | Uncharacterized protein | LOC101798331 | -3.42 | 5.19E-05 |
| U3I0F9 | Pyruvate kinase | PKM | -3.28 | 1.94E-07 |
| U3IE74 | L-lactate dehydrogenase | LOC101799210 | -3.26 | 9.12E-07 |
| U3ISF5 | Glutamine synthetase | GLUL | -3.22 | 1.69E-06 |
| U3I073 | Uncharacterized protein | LOC101800641 | -3.20 | 2.07E-05 |
| U3IEZ9 | Calpastatin | CAST | -3.13 | 4.09E-02 |
| U3IFN5 | Villin 1 | VIL1 | -3.12 | 1.13E-08 |
| R0K2W7 | Glycerol-3-phosphate dehydrogenase 2 (Fragment) | GPD2 | -3.11 | 1.70E-07 |
| Q7ZYV1 | Sodium/potassium-transporting ATPase subunit alpha | ATP1A1 | -3.11 | 0.00E+00 |
| U3IFH4 | 6-phosphogluconate dehydrogenase, decarboxylating | PGD | -3.08 | 9.09E-06 |
| U3J4Z9 | Acyl-CoA synthetase long chain family member 5 | ACSL5 | -3.06 | 6.74E-09 |
| U3J1L1 | Glyceraldehyde-3-phosphate dehydrogenase | GAPDH | -3.02 | 9.49E-06 |
| U3I4P7 | Retinol binding protein 2 | RBP2 | -2.99 | 6.01E-03 |
| U3ILU2 | Phospholipase B1 | PLB1 | -2.99 | 3.40E-14 |
| R0L951 | 3'(2'), 5'-bisphosphate nucleotidase 1 (Fragment) | BPNT1 | -2.99 | 5.24E-04 |
| R0JKW0 | Cytochrome c (Fragment) | CYCS | -2.95 | 1.99E-02 |
| R0LDD0 | Membrane metalloendopeptidase (Fragment) | MME | -2.95 | 1.54E-07 |
| R0LDL0 | Glutathione S-transferase theta-1 (Fragment) | Anapl_06111 | -2.94 | 2.49E-04 |
| U3I9J5 | Uncharacterized protein | LOC101798568 | -2.93 | 8.69E-05 |
| U3I8D8 | Triosephosphate isomerase | TPI1 | -2.93 | 9.45E-05 |
| U3IQT7 | UTP--glucose-1-phosphate uridylyltransferase | UGP2 | -2.92 | 3.31E-05 |
| Q2LDE6 | Transthyretin | TTR | -2.91 | 2.80E-02 |
| U3J4G6 | UDP-N-acetylglucosamine pyrophosphorylase 1 | UAP1 | -2.91 | 2.01E-03 |
| U3J2C8 | Glycerophosphodiester phosphodiesterase domain containing 1 | GDPD1 | -2.90 | 1.10E-04 |
| U3IA60 | Malate dehydrogenase | MDH2 | -2.89 | 4.45E-08 |
| U3J3L7 | Protein disulfide-isomerase | P4HB | -2.80 | 1.12E-10 |
| R0L3F2 | Microsomal triglyceride transfer protein large subunit (Fragment) | Anapl_12362 | -2.78 | 5.73E-14 |
| U3J057 | Phosphatidylethanolamine binding protein 1 | PEBP1 | -2.77 | 1.21E-03 |
| U3ICJ6 | 3-hydroxybutyrate dehydrogenase 1 | BDH1 | -2.75 | 1.05E-03 |
| U3IXH5 | UDP-glucose 6-dehydrogenase | UGDH | -2.72 | 3.23E-05 |
| U3IKH5 | Agmatinase | AGMAT | -2.69 | 4.37E-02 |
| R0K9C7 | Ezrin-radixin-moesin-binding phosphoprotein 50 | Anapl_03621 | -2.67 | 2.26E-02 |
| U3J221 | Chloride intracellular channel protein | CLIC5 | -2.66 | 6.25E-06 |
| U3I604 | Plastin 1 | PLS1 | -2.65 | 3.22E-07 |
| U3ISG4 | NPC1 like intracellular cholesterol transporter 1 | NPC1L1 | -2.63 | 9.86E-06 |
| U3J7R2 | Integrin subunit alpha 6 | ITGA6 | -2.61 | 7.32E-04 |
| U3IWJ8 | Integrin beta | ITGB4 | -2.61 | 2.83E-03 |
| U3IDD2 | Kyphoscoliosis peptidase | KY | -2.60 | 2.03E-03 |
| U3IBL0 | Uncharacterized protein | N/A | -2.58 | 7.44E-03 |
| U3IGV0 | Tubulin alpha chain | TUBAL3 | -2.57 | 2.20E-02 |
| R0L2L4 | Angiotensin-converting enzyme (Fragment) | Anapl_11772 | -2.57 | 3.93E-14 |
| U3J0T0 | Amine oxidase | MAOA | -2.56 | 5.04E-08 |
| R0LVZ8 | Cubilin (Fragment) | Anapl_16458 | -2.56 | 4.10E-02 |
| U3J6L3 | Voltage dependent anion channel 1 | VDAC1 | -2.55 | 2.74E-03 |
| U3IMR3 | Uncharacterized protein | PDZK1 | -2.54 | 2.25E-10 |
| U3J6M0 | Guanine deaminase | GDA | -2.54 | 3.17E-07 |
| U3I7Q9 | Uncharacterized protein | LOC101792912 | -2.53 | 1.38E-05 |
| U3ID33 | NADPH--cytochrome P450 reductase | POR | -2.52 | 1.16E-03 |
| R0J6N0 | Erythronolide synthase, modules 3 and 4 (Fragment) | Anapl_18411 | -2.52 | 1.04E-03 |
| R0K642 | Ladinin-1 | Anapl_02084 | -2.51 | 2.63E-03 |
| U3ID14 | Uncharacterized protein | N/A | -2.47 | 1.25E-03 |
| U3I5Q8 | IQ motif containing GTPase activating protein 2 | IQGAP2 | -2.46 | 3.82E-08 |
| U3IM27 | Uncharacterized protein | ALDH2 | -2.45 | 3.56E-05 |
| U3IS89 | Cytidine/uridine monophosphate kinase 1 | CMPK1 | -2.44 | 7.87E-03 |
| U3J2H8 | Fructose-bisphosphatase 1 | FBP1 | -2.42 | 1.35E-10 |
| U3I646 | Carbonic anhydrase 2 | CA2 | -2.42 | 2.11E-04 |
| U3ILX9 | Solute carrier family 3 member 1 | SLC3A1 | -2.38 | 2.61E-03 |
| R0KXP8 | Cadherin-17 (Fragment) | Anapl_13255 | -2.37 | 3.67E-05 |
| U3IIX3 | Rab GDP dissociation inhibitor | GDI2 | -2.35 | 6.10E-04 |
| U3IZA5 | ATP-dependent 6-phosphofructokinase | PFKP | -2.33 | 3.93E-05 |
| U3J919 | Lactase | LCT | -2.32 | 1.46E-07 |
| U3IH05 | Uncharacterized protein | FAM234A | -2.32 | 2.71E-02 |
| R0LMI1 | ES1 protein-like protein, mitochondrial (Fragment) | Anapl_08449 | -2.28 | 4.16E-02 |
| U3IUS6 | Monoacylglycerol O-acyltransferase 2 | MOGAT2 | -2.28 | 5.35E-03 |
| U3IZY2 | Fatty acid binding protein 2 | FABP2 | -2.28 | 1.26E-02 |
| U3I252 | ADP ribosylation factor guanine nucleotide exchange factor 1 | ARFGEF1 | -2.25 | 4.70E-02 |
| U3IA01 | Hematopoietic prostaglandin D synthase | HPGDS | -2.20 | 7.77E-05 |
| R0JQZ8 | Acyl-coenzyme A thioesterase 4 (Fragment) | Anapl_15305 | -2.17 | 5.07E-03 |
| R0LHP1 | Hydroxyacyl-coenzyme A dehydrogenase, mitochondrial (Fragment) | Anapl_04781 | -2.16 | 4.16E-03 |
| U3ITY7 | Uncharacterized protein | N/A | -2.15 | 1.20E-02 |
| U3IAQ2 | Meprin A subunit | MEP1A | -2.15 | 2.79E-10 |
| U3IYB6 | Aminopeptidase | ENPEP | -2.15 | 3.76E-09 |
| U3IC15 | Aconitate hydratase, mitochondrial | ACO2 | -2.13 | 9.22E-11 |
| U3J4X1 | Heat shock protein 90 beta family member 1 | HSP90B1 | -2.11 | 1.14E-03 |
| R0K1I7 | 55 kDa erythrocyte membrane protein (Fragment) | MPP1 | -2.05 | 1.26E-05 |
| U3IRT7 | Abhydrolase domain containing 6 | ABHD6 | -2.04 | 5.65E-03 |
| U3J8Q5 | Uncharacterized protein | N/A | -2.03 | 4.27E-03 |
| U3J4Y7 | CD2 associated protein | CD2AP | -2.02 | 1.43E-04 |
| U3ITQ4 | Sushi domain containing 2 | SUSD2 | -2.02 | 1.95E-02 |
| R0KRH1 | Zinc finger ZZ-type and EF-hand domain-containing protein 1 (Fragment) | Anapl_10240 | -2.02 | 1.06E-05 |
| R0M042 | Band 4.1-like protein 3 (Fragment) | Anapl_06632 | -2.02 | 8.44E-03 |
| U3ILF5 | Phosphoglycerate kinase | PGK1 | -2.01 | 1.71E-06 |
| R0JHT9 | Zinc transporter 10 (Fragment) | Anapl_13171 | -2.01 | 4.13E-02 |
| U3J1N4 | Calcineurin like EF-hand protein 1 | CHP1 | -2.00 | 1.68E-02 |
| U3IY96 | Ezrin | EZR | -2.00 | 1.61E-05 |
| R0KUF0 | Myosin-Ia | Anapl_15000 | -2.00 | 1.33E-02 |
| R0LHJ8 | Clathrin heavy chain 1 (Fragment) | Anapl_03930 | -1.99 | 9.98E-07 |
| R0JFQ9 | Peroxiredoxin-6 (Fragment) | PRDX6 | -1.99 | 8.62E-04 |
| U3I640 | Heat shock protein family A (Hsp70) member 5 | HSPA5 | -1.98 | 1.01E-04 |
| U3J7G5 | Heme binding protein 1 | HEBP1 | -1.97 | 4.00E-03 |
| R0K2Z3 | GDH/6PGL endoplasmic bifunctional protein (Fragment) | Anapl_11912 | -1.96 | 1.32E-04 |
| U3I9A1 | Acyl-CoA dehydrogenase family member 11 | ACAD11 | -1.96 | 1.34E-02 |
| U3IBV7 | Myosin ID | MYO1D | -1.94 | 1.57E-08 |
| U3I888 | Lissencephaly-1 homolog | PAFAH1B1 | -1.94 | 9.83E-03 |
| R0LKR3 | Glucose-6-phosphate isomerase (Fragment) | Anapl_00389 | -1.94 | 3.25E-04 |
| U3IKU2 | Sterol carrier protein 2 | SCP2 | -1.94 | 9.02E-07 |
| R0LSN9 | Myosin-VI (Fragment) | Anapl_03234 | -1.92 | 5.74E-05 |
| U3J7U1 | Ectonucleotide pyrophosphatase/phosphodiesterase 6 | ENPP6 | -1.90 | 4.21E-04 |
| U3ICI9 | Uncharacterized protein | HEPH | -1.90 | 2.61E-05 |
| U3IMG5 | USH1 protein network component harmonin | USH1C | -1.89 | 4.93E-02 |
| U3INW1 | Sideroflexin | SFXN1 | -1.89 | 4.65E-02 |
| U3IYF3 | Voltage dependent anion channel 2 | VDAC2 | -1.88 | 2.75E-02 |
| U3J9C7 | Solute carrier family 25 member 1 | SLC25A1 | -1.88 | 4.07E-03 |
| U3I8F1 | Glucosamine-6-phosphate isomerase | GNPDA1 | -1.88 | 2.77E-03 |
| R0J8F7 | Citrate lyase beta like (Fragment) | CLYBL | -1.87 | 2.35E-02 |
| U3ISD2 | Caspase 6 | CASP6 | -1.87 | 2.24E-03 |
| U3I004 | Chloride intracellular channel protein | CLIC4 | -1.86 | 1.21E-02 |
| U3IDA3 | Uncharacterized protein | LY75 | -1.86 | 8.29E-05 |
| U3I9L2 | Uncharacterized protein | N/A | -1.86 | 1.58E-02 |
| U3ITA9 | Uncharacterized protein | ACADM | -1.85 | 1.79E-05 |
| U3I535 | Aldo-keto reductase family 1 member D1 | AKR1D1 | -1.85 | 6.61E-07 |
| U3I509 | 15-hydroxyprostaglandin dehydrogenase | HPGD | -1.84 | 1.67E-02 |
| A0A172QNN4 | Catalase | CAT | -1.84 | 1.49E-05 |
| U3I693 | Acetyl-coenzyme A synthetase | ACSS1 | -1.83 | 3.99E-03 |
| U3INF1 | Mitochondrial calcium uniporter | MCU | -1.83 | 1.71E-02 |
| R0JRM9 | Putative E3 ubiquitin-protein ligase HECTD3 (Fragment) | Anapl_16663 | -1.83 | 1.30E-04 |
| U3IMG8 | Adenosine deaminase | ADA | -1.82 | 1.53E-06 |
| U3ISZ7 | Uncharacterized protein | LOC101805284 | -1.82 | 6.69E-03 |
| U3IWF6 | Uncharacterized protein | LOC101804020 | -1.81 | 2.45E-04 |
| U3IB11 | Nicalin | NCLN | -1.80 | 2.60E-02 |
| U3J213 | Atlastin GTPase 2 | ATL2 | -1.80 | 4.78E-02 |
| U3IYV2 | Aldose 1-epimerase | GALM | -1.80 | 2.18E-04 |
| U3IKI2 | Ectonucleotide pyrophosphatase/phosphodiesterase 7 | ENPP7 | -1.80 | 5.70E-03 |
| U3IAV6 | Aldehyde dehydrogenase 1 family member A1 | ALDH1A1 | -1.80 | 3.56E-08 |
| R0LIL9 | ATP synthase subunit O, mitochondrial (Fragment) | ATP5O | -1.80 | 2.12E-03 |
| U3J562 | Phosphate cytidylyltransferase 2, ethanolamine | PCYT2 | -1.79 | 6.41E-03 |
| U3J175 | ATP synthase, H+ transporting, mitochondrial Fo complex subunit B1 | ATP5F1 | -1.79 | 2.69E-04 |
| R0LRM7 | Destrin (Fragment) | DSTN | -1.79 | 3.59E-03 |
| R0JXM5 | Malate dehydrogenase (Fragment) | MDH1 | -1.78 | 2.25E-05 |
| U3IHS8 | Carnitine O-acetyltransferase | CRAT | -1.78 | 6.54E-03 |
| R0KWZ2 | Spectrin beta chain, brain 4 | Anapl_15190 | -1.78 | 4.06E-08 |
| U3ITV1 | Non-specific serine/threonine protein kinase | RPS6KA1 | -1.78 | 4.98E-02 |
| U3I6I0 | Adenosylhomocysteinase | AHCYL1 | -1.77 | 1.13E-05 |
| U3IUG2 | Plastin 3 | PLS3 | -1.77 | 2.12E-05 |
| U3INE4 | Carnosine dipeptidase 2 | CNDP2 | -1.76 | 8.43E-10 |
| R0M2K0 | Putative myosin-XVB (Fragment) | Anapl_03650 | -1.76 | 1.14E-03 |
| U3J236 | Glyoxalase domain containing 5 | GLOD5 | -1.76 | 6.18E-05 |
| U3IDQ1 | Acyl-coenzyme A oxidase | ACOX2 | -1.74 | 3.50E-05 |
| U3I5D2 | Leukotriene A(4) hydrolase | LTA4H | -1.74 | 1.25E-05 |
| U3ICS9 | Uncharacterized protein | N/A | -1.73 | 1.57E-03 |
| U3I3W9 | Crystallin lambda 1 | CRYL1 | -1.73 | 3.84E-05 |
| U3IIZ2 | Uncharacterized protein | N/A | -1.72 | 1.08E-02 |
| U3J8E3 | Nudix hydrolase 1 | NUDT1 | -1.72 | 1.28E-02 |
| U3ILP7 | Peptidase D | PEPD | -1.72 | 1.46E-08 |
| R0JU81 | Carboxylic ester hydrolase (Fragment) | Anapl_00395 | -1.72 | 1.55E-02 |
| U3IEP3 | Fatty acid binding protein 4 | FABP4 | -1.71 | 1.60E-02 |
| U3IUX5 | Uncharacterized protein | N/A | -1.71 | 4.30E-02 |
| R0KN24 | X-prolyl aminopeptidase 1 (Fragment) | XPNPEP1 | -1.70 | 3.93E-09 |
| U3J3I1 | Sodium/hydrogen exchanger | SLC9A3 | -1.69 | 4.10E-03 |
| R0JXF2 | Putative ATP-dependent RNA helicase DDX60 (Fragment) | Anapl_12150 | -1.69 | 2.45E-04 |
| U3II47 | Aldo-keto reductase family 1 member A1 | AKR1A1 | -1.68 | 6.23E-04 |
| U3IRM5 | Uncharacterized protein | LOC101803902 | -1.68 | 3.09E-04 |
| U3IX02 | Uncharacterized protein | N/A | -1.68 | 3.95E-02 |
| U3ILA4 | Prostaglandin reductase 1 | PTGR1 | -1.67 | 1.17E-03 |
| U3J7I2 | Glutathione synthetase | GSS | -1.67 | 1.42E-02 |
| U3IR48 | Dihydrolipoyl dehydrogenase | DLD | -1.66 | 2.09E-02 |
| U3I946 | Arp2/3 complex 34 kDa subunit | ARPC2 | -1.66 | 2.73E-02 |
| U3J1S2 | Polypeptide N-acetylgalactosaminyltransferase | GALNT6 | -1.66 | 3.71E-03 |
| R0KS10 | Serine/threonine-protein kinase 3 (Fragment) | Anapl_03883 | -1.66 | 2.66E-02 |
| R0JMV5 | Bleomycin hydrolase (Fragment) | BLMH | -1.65 | 2.95E-02 |
| U3INV9 | ATP binding cassette subfamily D member 3 | ABCD3 | -1.65 | 1.16E-02 |
| U3IRX8 | SEC31 homolog A, COPII coat complex component | SEC31A | -1.65 | 2.52E-04 |
| U3I6X7 | Uncharacterized protein | LETM1 | -1.65 | 2.57E-03 |
| U3IEF4 | Enoyl-CoA delta isomerase 2 | ECI2 | -1.65 | 4.61E-02 |
| U3I6S1 | Uncharacterized protein | HADHB | -1.65 | 1.60E-03 |
| R0J775 | Aconitase 1 (Fragment) | ACO1 | -1.64 | 2.75E-10 |
| R0LNM9 | Pyridoxal-dependent decarboxylase domain-containing protein 1 (Fragment) | Anapl_00953 | -1.63 | 1.75E-02 |
| R0L6N6 | Protein disulfide-isomerase (Fragment) | PDIA3 | -1.63 | 8.44E-07 |
| U3IDY5 | Uncharacterized protein | N/A | -1.62 | 1.85E-02 |
| R0KZF5 | Coatomer subunit alpha (Fragment) | Anapl_15943 | -1.62 | 2.74E-02 |
| U3I342 | Ubiquinol-cytochrome c reductase core protein 2 | UQCRC2 | -1.61 | 6.16E-04 |
| R0LDH3 | EH domain containing 3 (Fragment) | EHD3 | -1.61 | 4.65E-05 |
| U3INT4 | Dipeptidyl peptidase 4 | DPP4 | -1.61 | 1.26E-02 |
| R0LFU7 | UDP-glucuronosyltransferase 1-1 (Fragment) | Anapl_12379 | -1.61 | 6.20E-04 |
| U3IP65 | Uncharacterized protein | N/A | -1.61 | 6.22E-03 |
| R0LYJ7 | ATP synthase subunit d, mitochondrial (Fragment) | ATP5H | -1.60 | 2.16E-03 |
| U3IVL6 | Uncharacterized protein | N/A | -1.60 | 2.42E-02 |
| U3IR67 | Hydroxysteroid 17-beta dehydrogenase 4 | HSD17B4 | -1.59 | 2.44E-05 |
| R0LD68 | Adenosylhomocysteinase (Fragment) | Anapl_02415 | -1.59 | 1.70E-03 |
| R0JP64 | Heat shock 70 kDa protein 4 (Fragment) | Anapl_15153 | -1.59 | 2.30E-03 |
| U3J6P9 | ATPase H+ transporting V1 subunit D | ATP6V1D | -1.59 | 3.84E-02 |
| U3IWQ6 | Adenylyl cyclase-associated protein | CAP1 | -1.59 | 5.25E-03 |
| U3ITW0 | Deoxyribose-phosphate aldolase | DERA | -1.59 | 1.28E-02 |
| U3I1M8 | Sulfurtransferase | MPST | -1.59 | 9.12E-03 |
| U3IPY8 | Uncharacterized protein | LOC101796074 | -1.59 | 9.72E-05 |
| U3ITK2 | EPS8 like 2 | EPS8L2 | -1.58 | 1.26E-02 |
| U3IEW2 | Pyruvate dehydrogenase E1 beta subunit | PDHB | -1.58 | 1.79E-02 |
| U3J532 | NADH:ubiquinone oxidoreductase subunit A5 | NDUFA5 | -1.58 | 1.94E-02 |
| R0J6J5 | Butyrophilin subfamily 3 member A2 (Fragment) | Anapl_18621 | -1.57 | 4.63E-02 |
| R0JZP2 | Microsomal glutathione S-transferase 1 (Fragment) | MGST1 | -1.57 | 2.66E-02 |
| U3IST1 | Myosin VIIB | MYO7B | -1.57 | 3.82E-04 |
| R0LLX6 | NADH dehydrogenase [ubiquinone] 1 alpha subcomplex subunit 6 (Fragment) | NDUFA6 | -1.56 | 4.97E-02 |
| U3ILW3 | Uncharacterized protein | AARS | -1.55 | 1.94E-02 |
| U3J5H0 | Chromosome 11 open reading frame 54 | C11orf54 | -1.55 | 2.83E-05 |
| U3ISI9 | Transaldolase | TALDO1 | -1.55 | 5.04E-05 |
| U3J8E6 | Uncharacterized protein | LOC101794661 | -1.55 | 6.93E-05 |
| R0KF96 | Cytochrome P450 4V2 (Fragment) | CYP4V2 | -1.54 | 1.07E-02 |
| U3IXU4 | Acyl-CoA-binding domain-containing protein 5 | ACBD5 | -1.54 | 4.22E-02 |
| U3J6K2 | SEC31 homolog B, COPII coat complex component | SEC31B | -1.54 | 2.92E-02 |
| R0KPF7 | Inorganic pyrophosphatase | Anapl_06672 | -1.53 | 1.33E-03 |
| R0M5X6 | Aminopeptidase like 1 (Fragment) | NPEPL1 | -1.53 | 2.86E-04 |
| R0JXK4 | Ankyrin repeat and SAM domain-containing protein 4B (Fragment) | ANKS4B | -1.53 | 2.45E-02 |
| R0JV14 | Carboxylic ester hydrolase (Fragment) | Anapl_00396 | -1.53 | 1.85E-04 |
| U3IHZ5 | Short chain dehydrogenase/reductase family 16C member 5 | SDR16C5 | -1.52 | 2.16E-02 |
| R0L2P0 | LIM domain and actin-binding protein 1 (Fragment) | Anapl_13206 | -1.51 | 2.76E-02 |
| U3J928 | Acyl-coenzyme A oxidase | ACOX1 | -1.51 | 1.68E-05 |
| R0JV77 | Sulfide:quinone oxidoreductase, mitochondrial (Fragment) | SQOR | -1.51 | 2.57E-02 |
| U3I8M2 | Interferon induced protein 35 | IFI35 | -1.51 | 4.45E-03 |
| U3J667 | Uncharacterized protein | N/A | 1.50 | 2.63E-02 |
| U3ITE5 | Lectin, mannose binding 2 | LMAN2 | 1.51 | 1.12E-02 |
| R0JE14 | 60S ribosomal protein L23 (Fragment) | RPL23 | 1.51 | 1.18E-02 |
| R0M163 | von Willebrand factor (Fragment) | Anapl_04024 | 1.51 | 4.15E-02 |
| R0LG86 | Small nuclear ribonucleoprotein U5 subunit 200 (Fragment) | SNRNP200 | 1.52 | 1.94E-02 |
| U3I3X8 | Annexin | ANXA11 | 1.52 | 9.51E-03 |
| U3J5E2 | S-(hydroxymethyl)glutathione dehydrogenase | ADH5 | 1.52 | 6.32E-05 |
| U3I3K4 | Tight junction protein 2 | TJP2 | 1.53 | 2.18E-02 |
| U3J3R0 | Uncharacterized protein | DDX3X | 1.53 | 1.05E-02 |
| R0LPL6 | Asparagine synthetase (glutamine-hydrolyzing) (Fragment) | ASNS | 1.53 | 1.19E-02 |
| U3I418 | Uncharacterized protein | N/A | 1.53 | 4.88E-02 |
| R0L492 | Nuclear pore complex protein Nup107 (Fragment) | Anapl_07419 | 1.54 | 2.38E-02 |
| R0LDG6 | Histamine N-methyltransferase (Fragment) | HNMT | 1.54 | 3.32E-03 |
| U3J491 | Serine and arginine rich splicing factor 6 | SRSF6 | 1.55 | 4.44E-02 |
| U3J6X7 | Lamin B1 | LMNB1 | 1.55 | 4.05E-02 |
| U3IE28 | Heterochromatin protein 1 binding protein 3 | HP1BP3 | 1.56 | 2.30E-03 |
| U3IIY7 | Uncharacterized protein | LOC101799795 | 1.56 | 1.55E-04 |
| U3J8J5 | Galectin | LGALS2 | 1.57 | 1.59E-03 |
| R0M4G2 | Cell division cycle and apoptosis regulator protein 1 (Fragment) | Anapl_06172 | 1.57 | 2.94E-02 |
| R0K419 | Heme oxygenase 1 (Fragment) | Anapl_09648 | 1.57 | 1.61E-02 |
| U3I5T1 | Glutamate-cysteine ligase catalytic subunit | GCLC | 1.57 | 9.62E-03 |
| U3J2Z0 | Heterogeneous nuclear ribonucleoprotein A/B | HNRNPAB | 1.57 | 4.81E-03 |
| R0LH17 | Splicing factor 3B subunit 1 (Fragment) | Anapl_04559 | 1.57 | 9.97E-04 |
| U3J597 | Isocitrate dehydrogenase [NADP] | IDH1 | 1.58 | 5.05E-06 |
| R0LQS4 | Calpain-1 catalytic subunit (Fragment) | Anapl_05703 | 1.58 | 9.53E-03 |
| U3IWZ0 | Ribosomal protein S13 | RPS13 | 1.59 | 1.53E-02 |
| U3I7T9 | Acyl-CoA synthetase family member 2 | ACSF2 | 1.59 | 3.23E-02 |
| U3I937 | Amine oxidase | N/A | 1.62 | 4.34E-02 |
| R0L459 | LEM domain containing 2 (Fragment) | LEMD2 | 1.63 | 4.46E-02 |
| U3IEQ8 | Sjogren syndrome antigen B | SSB | 1.64 | 5.73E-03 |
| R0KEZ2 | Phenazine biosynthesis-like domain-containing protein (Fragment) | Anapl_06179 | 1.64 | 1.76E-02 |
| U3INY7 | Uncharacterized protein | RNH1 | 1.65 | 8.46E-04 |
| R0LC19 | Heat shock 70 kDa protein (Fragment) | HSPA2 | 1.65 | 1.63E-02 |
| U3I0C5 | Lymphocyte cytosolic protein 1 | LCP1 | 1.66 | 7.73E-03 |
| U3IS69 | Uncharacterized protein | HNRNPH1 | 1.66 | 4.23E-02 |
| U3J2L5 | Uncharacterized protein | IPO5 | 1.67 | 1.57E-02 |
| U3IZF2 | Translocated promoter region, nuclear basket protein | TPR | 1.67 | 6.39E-05 |
| U3IUB7 | Lamin B2 | LMNB2 | 1.67 | 1.73E-02 |
| U3J730 | Ornithine aminotransferase | OAT | 1.68 | 1.79E-02 |
| U3J8Q3 | Regulator of chromosome condensation 2 | RCC2 | 1.69 | 2.83E-02 |
| U3J4P7 | Ribosomal protein L8 | RPL8 | 1.69 | 3.15E-02 |
| R0LN18 | Spectrin beta chain, brain 1 (Fragment) | SPTBN1 | 1.69 | 1.25E-09 |
| U3IDY0 | Coenzyme Q9 | COQ9 | 1.69 | 2.33E-02 |
| U3I6K3 | Uncharacterized protein | N/A | 1.69 | 1.78E-04 |
| R0JDR4 | Pterin-4 alpha-carbinolamine dehydratase 1 (Fragment) | PCBD1 | 1.70 | 3.71E-02 |
| R0L7C1 | Propionyl-CoA carboxylase beta chain, mitochondrial (Fragment) | PCCB | 1.70 | 4.23E-02 |
| U3I8U0 | Uncharacterized protein | N/A | 1.70 | 2.09E-04 |
| U3I8L1 | Exportin 1 | XPO1 | 1.71 | 2.11E-02 |
| U3J1N9 | GTP-binding nuclear protein Ran | RAN | 1.72 | 4.34E-02 |
| U3IHY5 | Damage specific DNA binding protein 1 | DDB1 | 1.73 | 2.11E-03 |
| U3IPW4 | Uncharacterized protein | N/A | 1.73 | 5.06E-03 |
| U3J4D8 | Prefoldin subunit 3 | VBP1 | 1.73 | 2.06E-02 |
| U3J5N5 | Prolyl endopeptidase | PREP | 1.74 | 1.91E-04 |
| U3IP13 | SWI/SNF related, matrix associated, actin dependent regulator of chromatin, subfamily a, member 5 | SMARCA5 | 1.74 | 7.80E-03 |
| U3IZU3 | Family with sequence similarity 120A | FAM120A | 1.75 | 1.14E-02 |
| R0JE94 | Uncharacterized protein (Fragment) | LOC101794704 | 1.75 | 1.17E-03 |
| R0LMH6 | RNA transcription, translation and transport factor (Fragment) | RTRAF | 1.75 | 2.90E-02 |
| U3J3C9 | Poly [ADP-ribose] polymerase | PARP1 | 1.76 | 2.64E-02 |
| U3I5Y3 | Programmed cell death 4 | PDCD4 | 1.76 | 5.73E-03 |
| U3IS14 | Coproporphyrinogen oxidase | CPOX | 1.77 | 2.76E-02 |
| U3IJU8 | Aldehyde dehydrogenase 8 family member A1 | ALDH8A1 | 1.77 | 2.58E-02 |
| U3IN65 | Plectin | PLEC | 1.77 | 0.00E+00 |
| R0LHG2 | Putative RNA-binding protein 25 (Fragment) | Anapl_01114 | 1.77 | 4.32E-02 |
| R0LJ35 | Heterogeneous nuclear ribonucleoprotein A3 (Fragment) | Anapl_04750 | 1.78 | 7.12E-03 |
| R0L5I6 | Nuclear pore complex protein Nup133 (Fragment) | Anapl_05253 | 1.80 | 4.30E-02 |
| R0L173 | Gelsolin (Fragment) | Anapl_16652 | 1.80 | 5.46E-03 |
| U3I341 | Histone H4 | HIST1H4D | 1.82 | 2.31E-05 |
| R0LE95 | Alpha-1,4 glucan phosphorylase (Fragment) | Anapl_16320 | 1.83 | 4.65E-05 |
| R0K9F9 | GMP synthase [glutamine-hydrolyzing] (Fragment) | GMPS | 1.84 | 4.56E-02 |
| U3IFC3 | Uncharacterized protein | LOC101794821 | 1.85 | 4.64E-02 |
| U3IX39 | Heterogeneous nuclear ribonucleoprotein A2/B1 | HNRNPA2B1 | 1.86 | 1.06E-02 |
| U3IR26 | Vinculin | VCL | 1.87 | 4.54E-11 |
| U3IYG4 | Uncharacterized protein | N/A | 1.88 | 3.24E-02 |
| R0KGA6 | Glutathione transferase omega-1 (Fragment) | Anapl_11100 | 1.88 | 1.55E-05 |
| R0KCY9 | Desmoplakin (Fragment) | Anapl_03788 | 1.89 | 1.24E-06 |
| U3I5N9 | Switching B-cell complex subunit SWAP70 | SWAP70 | 1.89 | 3.89E-02 |
| R0JBU7 | Methionine adenosyltransferase 2 subunit beta (Fragment) | MAT2B | 1.89 | 1.82E-02 |
| U3I939 | Fructose-bisphosphate aldolase | ALDOA | 1.89 | 1.34E-03 |
| U3J4E5 | Ribosomal protein S3 | RPS3 | 1.90 | 2.25E-02 |
| U3HZR3 | Uncharacterized protein | N/A | 1.91 | 3.56E-04 |
| U3I4U5 | Erbb2 interacting protein | ERBIN | 1.91 | 9.63E-03 |
| U3J207 | Transformer 2 alpha homolog | N/A | 1.93 | 3.27E-02 |
| U3IXM0 | Peptidylprolyl isomerase | FKBP4 | 1.94 | 2.79E-05 |
| U3I536 | Ankyrin repeat domain 44 | ANKRD44 | 1.95 | 3.85E-02 |
| U3IHU7 | Macrophage expressed 1 | MPEG1 | 1.95 | 3.36E-02 |
| U3I7Q0 | Heterogeneous nuclear ribonucleoprotein R | HNRNPR | 1.96 | 4.45E-03 |
| U3J3X9 | 5-aminoimidazole-4-carboxamide ribonucleotide formyltransferase/IMP cyclohydrolase | ATIC | 1.96 | 2.11E-04 |
| U3ISZ1 | Uncharacterized protein | LOC101798161 | 1.97 | 3.83E-02 |
| R0KAG8 | Cellular nucleic acid-binding protein (Fragment) | Anapl_09071 | 1.98 | 3.06E-02 |
| U3III1 | Uncharacterized protein | N/A | 1.98 | 1.24E-02 |
| U3J4U7 | Uncharacterized protein | N/A | 1.99 | 4.90E-02 |
| U3ILA0 | Uncharacterized protein | PA2G4 | 1.99 | 1.51E-03 |
| U3IBF3 | RAB3 GTPase activating non-catalytic protein subunit 2 | RAB3GAP2 | 2.00 | 4.01E-02 |
| U3IVZ0 | Nitrilase family member 2 | NIT2 | 2.01 | 2.23E-02 |
| U3IC18 | Glutathione S-transferase kappa | GSTK1 | 2.02 | 3.24E-03 |
| U3ILL9 | Uncharacterized protein | RPL17 | 2.03 | 1.82E-02 |
| U3IJ39 | AHNAK nucleoprotein | AHNAK | 2.03 | 1.79E-11 |
| U3IJF7 | Desmoglein 2 | DSG2 | 2.05 | 2.87E-03 |
| R0JSM9 | Heat shock protein HSP 90-alpha (Fragment) | Anapl_00734 | 2.06 | 1.21E-09 |
| U3I935 | Moesin | MSN | 2.07 | 5.48E-03 |
| U3J4P9 | Ribosomal protein L3 | RPL3 | 2.07 | 3.88E-02 |
| R0LHT7 | Chromogranin-A (Fragment) | Anapl_03288 | 2.07 | 2.70E-02 |
| U3J044 | Uncharacterized protein | N/A | 2.08 | 4.27E-02 |
| U3I479 | Uncharacterized protein | N/A | 2.10 | 6.67E-03 |
| U3IRL7 | Uncharacterized protein | LOC101801727 | 2.12 | 1.07E-03 |
| U3I4I9 | Actinin alpha 1 | ACTN1 | 2.13 | 5.89E-07 |
| U3IXU2 | Heterogeneous nuclear ribonucleoprotein U | HNRNPU | 2.13 | 8.95E-04 |
| U3IAL5 | Plakophilin 2 | PKP2 | 2.13 | 3.96E-02 |
| U3IQU7 | Pre-mRNA processing factor 4B | PRPF4B | 2.13 | 3.52E-03 |
| U3IKI8 | Uncharacterized protein | N/A | 2.14 | 3.98E-09 |
| U3I4Q4 | Nucleoside diphosphate kinase | N/A | 2.15 | 1.56E-02 |
| U3J0Q0 | Acyl-CoA binding domain containing 3 | ACBD3 | 2.17 | 3.56E-02 |
| R0KF66 | STIP1 homology and U-box containing protein 1 (Fragment) | STUB1 | 2.18 | 1.48E-02 |
| P04442 | Hemoglobin subunit alpha-D | HBAD | 2.20 | 4.46E-02 |
| U3IWS8 | Uncharacterized protein | N/A | 2.20 | 1.24E-03 |
| R0KFP7 | Glutathione S-transferase (Fragment) | LOC101797566 | 2.21 | 2.21E-05 |
| U3IRD0 | Uncharacterized protein | N/A | 2.23 | 4.39E-02 |
| U3J0T9 | Insulin like growth factor 2 mRNA binding protein 3 | IGF2BP3 | 2.26 | 3.98E-02 |
| Q1HFX7 | MHC class II antigen beta chain | N/A | 2.26 | 1.61E-02 |
| U3IZ83 | Fibronectin 1 | FN1 | 2.28 | 4.31E-02 |
| U3J9D5 | Endothelial differentiation related factor 1 | EDF1 | 2.31 | 4.69E-02 |
| U3J8Z8 | Proliferating cell nuclear antigen | PCNA | 2.35 | 1.57E-02 |
| U3IX14 | Thymocyte nuclear protein 1 | THYN1 | 2.40 | 3.76E-02 |
| R0LBU5 | Dihydropyrimidinase like 2 (Fragment) | DPYSL2 | 2.42 | 4.44E-02 |
| U3IYU3 | Transglutaminase 4 | TGM4 | 2.48 | 7.89E-04 |
| U3IZC0 | LIM domain containing preferred translocation partner in lipoma | LPP | 2.48 | 7.92E-03 |
| R0LY36 | Histone H3 (Fragment) | LOC101798542 | 2.48 | 1.19E-02 |
| U3ITK6 | Cystathionine gamma-lyase | CTH | 2.52 | 2.17E-02 |
| R0JSN7 | Histone-lysine N-methyltransferase (Fragment) | Anapl_00767 | 2.54 | 2.98E-02 |
| U3I572 | Polyribonucleotide nucleotidyltransferase 1 | PNPT1 | 2.55 | 1.67E-03 |
| U3J1D2 | TATA element modulatory factor 1 | TMF1 | 2.59 | 3.80E-02 |
| U3IEV9 | BAI1 associated protein 2 like 1 | BAIAP2L1 | 2.60 | 1.89E-02 |
| U3IV21 | Far upstream element binding protein 3 | FUBP3 | 2.61 | 1.13E-02 |
| U3ID73 | Uncharacterized protein | RPL7 | 2.62 | 8.46E-06 |
| U3IRY0 | Vimentin | VIM | 2.66 | 5.54E-04 |
| U3ISE5 | Chromosome segregation 1 like | CSE1L | 2.69 | 9.68E-04 |
| U3IBP5 | Uncharacterized protein | LOC101804845 | 2.74 | 3.71E-02 |
| U3J7M7 | Tetraspanin | TSPAN8 | 2.75 | 8.99E-04 |
| U3IRX9 | Nucleophosmin 1 | NPM1 | 2.82 | 6.19E-04 |
| R0LM85 | Myosin light chain kinase (Fragment) | MYLK | 2.88 | 1.83E-02 |
| R0LRR5 | Epidermal growth factor receptor kinase substrate 8 (Fragment) | Anapl_05586 | 2.91 | 5.53E-04 |
| R0KLK4 | 28S ribosomal protein S9, mitochondrial (Fragment) | Anapl_17837 | 2.93 | 1.87E-03 |
| U3IHI0 | Solute carrier family 12 member 2 | SLC12A2 | 2.96 | 2.65E-03 |
| R0JJW5 | Collagen alpha-2(VI) chain (Fragment) | Anapl_09440 | 2.97 | 8.85E-05 |
| U3IMH5 | Triacylglycerol lipase | PNLIP | 2.99 | 1.27E-02 |
| R0KHK4 | Protein FAM115C | Anapl_18320 | 3.03 | 4.59E-04 |
| U3I6R1 | Annexin | ANXA6 | 3.05 | 8.54E-08 |
| R0LPZ6 | Thioredoxin domain containing 5 (Fragment) | TXNDC5 | 3.09 | 1.28E-03 |
| R0KFX1 | Heterogeneous nuclear ribonucleoprotein H3 (Fragment) | HNRNPH3 | 3.19 | 4.45E-02 |
| U3IA23 | Fibrinogen gamma chain | FGG | 3.27 | 1.52E-02 |
| R0LY18 | Phospholipase B-like (Fragment) | Anapl_01056 | 3.31 | 3.49E-03 |
| U3IR68 | Hexokinase 1 | HK1 | 3.31 | 1.12E-02 |
| U3IES1 | Annexin | ANXA2 | 3.31 | 1.88E-13 |
| U3IXF6 | COMM domain containing 4 | COMMD4 | 3.36 | 4.48E-02 |
| U3IAF7 | Copine 1 | CPNE1 | 3.43 | 7.37E-04 |
| U3J9B8 | Uncharacterized protein | N/A | 3.46 | 2.26E-04 |
| U3IL65 | Nebulin | NEB | 3.59 | 7.12E-04 |
| U3I195 | Uncharacterized protein | N/A | 3.59 | 3.25E-02 |
| P09426 | Histone H1 | N/A | 3.60 | 3.36E-03 |
| U3IWW5 | Chromobox 3 | CBX3 | 3.71 | 2.51E-02 |
| U3IC54 | Uncharacterized protein | N/A | 3.78 | 1.61E-02 |
| C7EKN9 | Hemoglobin alpha A subunit | HBA2 | 3.89 | 3.56E-02 |
| U3J4M1 | Uncharacterized protein | N/A | 4.10 | 2.17E-02 |
| C7EMB6 | Hemoglobin beta A subunit | HBB | 4.19 | 3.76E-08 |
| U3IPP2 | Olfactomedin 4 | OLFM4 | 4.24 | 3.47E-06 |
| R0M884 | Glutathione S-transferase (Fragment) | LOC101798048 | 4.32 | 8.93E-04 |
| U3IJX3 | Hexosaminidase subunit beta | HEXB | 4.41 | 1.07E-02 |
| U3J6G2 | Tropomyosin 3 | TPM3 | 4.51 | 1.36E-02 |
| U3I4P0 | Sulfotransferase | LOC101801341 | 4.61 | 4.83E-03 |
| U3ITZ1 | Cytochrome P450 | LOC101790812 | 4.64 | 2.77E-02 |
| U3IHY7 | Ferritin | N/A | 4.86 | 2.17E-04 |
| U3IUK6 | Uncharacterized protein | N/A | 4.97 | 5.73E-03 |
| U3I4U2 | Transferrin receptor | TFRC | 4.99 | 1.45E-05 |
| U3HZV7 | Uncharacterized protein | LOC101798824 | 5.07 | 4.92E-05 |
| R0K6C9 | Titin (Fragment) | Anapl_04762 | 5.28 | 0.00E+00 |
| R0LDN0 | Deleted in malignant brain tumors 1 protein | Anapl_13855 | 5.33 | 5.04E-03 |
| R0M1Q9 | M-protein, striated muscle (Fragment) | Anapl_09328 | 5.54 | 2.73E-05 |
| R0JQ38 | Tenascin (Fragment) | Anapl_13701 | 5.63 | 7.61E-03 |
| U3ID88 | Collagen type VI alpha 3 chain | COL6A3 | 5.73 | 1.69E-04 |
| U3J1E5 | Uncharacterized protein | CLCA4 | 5.75 | 1.09E-08 |
| R0LV18 | Cysteine and glycine-rich protein 1 (Fragment) | Anapl_02081 | 6.00 | 9.55E-04 |
| R0JBP7 | Transgelin (Fragment) | TAGLN3 | 6.01 | 1.42E-02 |
| A0A1L5 | MHC class I antigen | Anpl-UAA | 6.08 | 7.52E-03 |
| U3J7T0 | Uncharacterized protein | N/A | 6.17 | 1.65E-03 |
| U3ILZ6 | Uncharacterized protein | N/A | 6.28 | 4.36E-03 |
| U3IGH1 | Creatine kinase, mitochondrial 2 | CKMT2 | 6.34 | 2.94E-02 |
| U3I3M1 | 3'-phosphoadenosine 5'-phosphosulfate synthase 2 | PAPSS2 | 6.44 | 5.77E-15 |
| U3IFD3 | Myosin heavy chain 11 | MYH11 | 6.83 | 1.16E-10 |
| U3IZP7 | Nucleolin | NCL | 7.36 | 1.16E-02 |
| R4HH66 | Desmin (Fragment) | N/A | 8.25 | 3.73E-03 |
| R0KZK3 | Tropomyosin alpha-1 chain (Fragment) | Anapl_05914 | 8.36 | 3.63E-03 |
| R0JKK6 | Collagen alpha-1(VI) chain (Fragment) | Anapl_09441 | 9.07 | 2.52E-04 |
| U3I7J8 | Uncharacterized protein | TPM1 | 9.58 | 6.76E-04 |
| U3IA79 | Myosin light chain 1 | MYL1 | 11.47 | 2.50E-06 |
| U3IWZ4 | Uncharacterized protein | N/A | 12.14 | 5.77E-06 |
| R0L9B8 | GEM-interacting protein (Fragment) | Anapl_10627 | 13.90 | 3.44E-02 |
| U3IX27 | Troponin T3, fast skeletal type | TNNT3 | 14.16 | 2.76E-03 |
| U3IEU8 | Myozenin 1 | MYOZ1 | 17.16 | 5.59E-03 |

* Fold change is expressed as the ratio of the pantothenic acid-deficient to the control group. For the downregulated proteins, the fold change was transformed to the corresponding negative value.
