# Supplementary material for: Severe pantothenic acid deficiency induces alterations in the intestinal mucosal proteome of starter Pekin ducks
Source: BMC Genomics. 2021 Jun 30;22:491. doi: 10.1186/s12864-021-07820-x (PMC8246668; doi:10.1186/s12864-021-07820-x)
Supplement: Supplementary file 2 — Additional file 2 Fig. S1. Complete images of Western blots shown in Fig. 3. Western blot analysis of medium-chain-specific acyl-CoA dehydrogenase (ACADM) and glyceraldehyde-3-phosphate dehydrogenase (GAPDH) protein abundance of mucosal tissue of ducks in the pantothenic acid deficient (PAD) and Control (CON) groups. Histone H3 served as a loading control. [file 12864_2021_7820_MOESM2_ESM.docx]

**Fig S1.** Complete images of Western blots shown in Figure 3. Western blot analysis of medium-chain-specific acyl-CoA dehydrogenase (ACADM) and glyceraldehyde-3-phosphate dehydrogenase (GAPDH) protein abundance of mucosal tissue of ducks in the pantothenic acid deficient (PAD) and Control (CON) groups. Histone H3 served as a loading control.
